# Exploring Knowledge about Fang Traditional Medicine: An Informal Health Seeking Behaviour for Medical or Cultural Afflictions in Equatorial Guinea

**DOI:** 10.3390/healthcare11060808

**Published:** 2023-03-09

**Authors:** Raquel Jimenez-Fernandez, Rocío Rodriguez Vázquez, Dolores Marín-Morales, Elena Herraiz-Soria, Marta Elena Losa-Iglesias, Ricardo Becerro-de-Bengoa-Vallejo, Inmaculada Corral-Liria

**Affiliations:** 1 Nursing and Dentistry Department, Faculty of Health Science, King Juan Carlos University, 28922 Madrid, Spain; 2 Faculty of Nursing, Physical Therapy and Podiatry, Complutense University of Madrid, 28040 Madrid, Spain

**Keywords:** Fang ethnic group, Equatorial Guinea, health-seeking behavior, traditional medicine, ethnomedicine, traditional healers

## Abstract

This study explores a range of informal health-seeking behaviors, including the use of Fang Traditional Medicine (FTM) for medical or cultural afflictions in Equatorial Guinea (EQ), the therapeutic methods used, the health problems handled, the learning process, traditional medicine user profiles and the social images of Fang Traditional Healers (FTHs). Ethnography was employed as a qualitative strategy using emic–etic approaches. Semi-structured interviews were conducted with 45 individuals, including 6 community leaders, 19 tribal elders, 7 healthcare professionals, 11 FTHs and 2 relatives of traditional healers in 5 districts of EQ. FTM offers a cure for malaria and treatments for reproductive health issues, bone fractures and cultural illnesses. Several methods used to learn FTM are based on empirical observation, and without the need for traditional schooling, unlike with Western medical professionals: for example, watching a family member, or the spirits or ancestors, can reveal healing knowledge. Materials from forests, including tree barks and plants, and rituals are used to keep Fang populations healthy; in addition, two rituals known as “*osuiñ*” and “*etoak*” (infusions of tree barks with the blood of sacrificed animals) are the most commonly used treatments. In addition, elders and women are the most active consumers of FTM. FTM plays a relevant role in curing medical and cultural afflictions in Fang communities. The informal health-seeking behavior among the Fang community is conditioned by the explanation model of illness.

## 1. Background

Approximately 80% of the rural population of Sub-Saharan Africa still uses medication therapies that are based on herbs, tree bark, parts of animals [1], minerals, and/or non-medication therapies in order to improve their health status [2,3,4]. According to the WHO, traditional medicine (TM) not only solves medical problems [5] related to the body and/or mind, but also tries to neutralize the negative factors that impede social balance or hamper individual advancement [6].

Traditional medicine practitioners tend to be the first ‘professionals’ consulted because they are more accessible geographically; indeed, they share the same villages and live in contiguous houses. The majority of them belong to their family and provide culturally accepted health practices or treatments that have been developed successfully since their ancestors. It allows them to maintain a high social status because of their local influence [7,8,9].

For the Fang ethnic group, who today live on Principe Island and in central and southwest Cameroon, Congo-Brazzaville, Gabon, and Equatorial Guinea [10], health, illness and death are manifestations of entities that appear as a consequence of sin, the breaking of laws of tradition, or through some malignant entity that acts to procure benefits for a third person [11]. The Fang are animists by definition; animals, plants, the elements, and inanimate objects can be a projection of the spirits of their ancestors [11,12].

Until 1985, Equatorial Guinea (EQ) did not officially recognize traditional medicine practices. At that time, Law 4/1985 was enacted, which created the National Directorate of Traditional Medicine, dependent on the Ministry of Health and Social Welfare. The Association of Traditional Medicine of Equatorial Guinea (ASOMETRAGE) now officially registers all Fang traditional healers (FTHs). However, EQ does not have procedures for the official approval of traditional medical practices or remedies. Traditional medicine practitioners are not involved in Equatorial Guinea’s primary healthcare program [5,8,13].

Some previous studies have argued that there are many kinds of African traditional healers [14,15,16,17,18] who work using different methods: for example, there are herbalists [19,20,21] or indigenous healers, and Pentecostal or Christian faith healers [22], who are in connection with the belief system [23] of the population, as well as the agent of the disease. Other lines of research have reported upon the experiences obtained in terms of healing that is related to specific areas: the treatment of HIV [24,25,26], mental illness, malaria, pregnancy or childbirth, fractures, etc. [27,28,29]. Recently, in addition to the WHO strategy, there have also been studies on how traditional medicine can be integrated into the biomedical system with excellent results, notably reducing mortality without any adverse effects on countries such as Ghana [30] or South Africa [31,32].

Therefore, the aim of the current study is to explore Fang traditional medicine (FTM), which is used as a therapeutic resource by a significant percentage of the Fang population in EQ; to discuss traditional medicine user profiles and the social image of Fang traditional healers (FTHs) among the Fang population; and to examine the health problems FTM addresses, as well as the FTM learning process, procedures and raw materials used to cure diseases through diverse Fang perspectives, including those of community leaders, tribal elders, hospital personnel, FTHs, and the relatives of traditional healers. Melding the cultural customs, safe practices and proper training of traditional medicine practitioners together, represent the greatest challenges for the WHO Traditional Medicine strategy (2014–2023) [33].

## 2. Methods

### 2.1. Study Area and Population

EQ, a country located in the Central African region, is divided into two administrative regions, the mainland and insular regions. According to data from 2015, EQ has a population of 845,000 people [13], with the Fang ethnic group being predominate (85.7%). The Fang mainly live in rural areas (62.2%) within tribal villages [34].

The United Nations Development Program (UNDP) evaluated Equatorial Guinea’s HDI value for 2018 as 0.588, positioning it at number 144 out of 189 countries and territories. EQ has a GDP per capita of USD 17.796 [35], and its total expenditure on health is 3.8% of its GDP [36], which is the largest gap in the world between INB and HDI. EQ registered a live births/maternal mortality ratio of 301/10,000 [37], and an under-five mortality rate of 94, 1/1000 in 2015; 68.3% of births are attended by skilled healthcare professionals, and there were 4.0 doctors and 9.3 hospital beds per 10,000 inhabitants in 2017 [34].

In 2004–2014, EQ experienced unprecedented economic growth based on the benefits of exploiting its oil and gas resources. As a result, the communications network, and educational and health facilities (hospitals and health centers) have been modernized [13,35]. Several models of the healthcare system are currently in place [38]: (1) a private healthcare system consisting of expatriate doctors and modern hospitals; (2) social and community health insurance, called “INSESO”, for Guinean employees; (3) a public healthcare system comprising health posts, health centers, 16 hospital districts and two regional hospitals located in Bata and Malabo, at which the government provides the professionals, but the treatment and diagnosis are paid for by the patients and their relatives out of pocket; (4) programs financed through Spanish (FERS) or North American religious organizations; (5) Chinese clinics; and (6) traditional healing posts [39].

### 2.2. Participant Recruitment

Purposeful and snowball sampling techniques were used to recruit participants. External facilitators who knew the rural population in each district, in collaboration with the mayors of the tribal villages, referred community leaders and tribal elders of the most representative clans in each area. Afterwards, the lucidity, eloquence and ability of each candidate in engaging in dialogue was evaluated by the research team. The inclusion criteria were as follows: belonging to the Fang ethnic group, living in the mainland region, being of EQ nationality, and being accessible to and well-known by at least 5 members of the community.

### 2.3. Participants

A total of 45 individuals of the Fang ethnic group (Table 1), belonging to 24 Fang clans (Table 2), were surveyed, including 6 community leaders, 19 tribal elders, 7 healthcare professionals, 11 FTHs and 2 relatives of traditional healers. None declined to participate in the study.

### 2.4. Data Collection

For the qualitative methodology, descriptive ethnography was used; this followed a previous 3-month Fang culture immersion project that used emic (from the perspective of the participants) and etic (from the perspective of the observer) approaches, as has been recommended [39,40,41,42].

This immersion phase, in the form of a “learning journey” in the Fang culture, included the following: training of the local team in qualitative research tools, including data collection, methods, analysis, etc.; and contacting a support team and renting a logistic base, in order to obtain travel and work permits in the villages, and explore the rural or urban context in which the daily life of the Fang population takes place, including churches, local markets, forests, hospitals, quackeries, etc. This allowed us to learn about their habits, rites, ceremonies, social structure, verbal expressions of feelings and how they behave regarding taboos, improper facts, forbidden witchcraft, idolatry, the ancestral Fang religion “melan”, or treatments for illness or death. Finally, we carried out a short pilot sample in order to amend some aspects of the study, such as the logical order or the comprehensibility of questions, and assess the feasibility and convenience of the field and subjects of study.

From September to December 2017, we conducted semi-structured interviews in 21/724 Fang tribal villages (Table 3) in 5/13 districts in the mainland region of EQ: Bata, Mongomo, Akonibe, Niefang and Kogo.

The sociodemographic profile of the participants (Table 4) was obtained using indicators (101–127) from the DHS [43] and was analyzed using IBM-SPSS Statistics, version 19.

Permission was gained from authorities to engage in immersion in the Fang tribal villages, select external facilitators and plan field activities [44]. The interviews were filmed, had an estimated duration between 10 and 40 min, and were conducted in either Fang or Spanish [45]. An interpreter led the interviews in the vernacular language.

### 2.5. Data Analysis

Semi structured interviews were transcribed from the recording, and the translation of Fang–Spanish was independently validated three times by the participants, the research team and, finally, by a Fang person who was not involved in the study.

The data obtained via the semi-structured interviews and participant observations were fragmented into the type of informant, collective, and the categories of analysis, which were generated from the dimensions of the interview script. Special emphasis was placed on the fragments that contained unique information or information that was repeated systematically [46]. Particularly meaningful data that were collected via interviews with participants of different ages, professional statuses, clans, and districts [45] were gathered. A qualitative methodology with an inductive focus and emergent character was used until disagreements were resolved [47].

Five thematic categories were identified (Table 5): (1) Specialties of FTM; (2) The FTM learning process; (3) Therapeutic FTM procedures for curing diseases; (4) Social image of FTHs among the Fang population; and (5) Traditional medicine user profiles.

Two members of the research team categorized the narratives separately and then compared the categorizations obtained. Categories and subcategories turned out to be very similar, and those that showed discrepancies were jointly reviewed and highlighted in the original recordings, in the presence of a third person. Key information on aspects of how to cure different ailments was often difficult to discern from the FTHs, as that knowledge is the essence of their business.

Finally, data were imported and analyzed with NVivo 9 Qualitative Data Analysis software (QSR International Pty Ltd. Cardigan UK) [48,49]. These are the data on which the conclusions are based.

The study adhered to COREQ guidelines [50]. Regarding Domain 1, the personal characteristics of the researcher, including their sex, credentials, occupation, experience in the field, as well as their prior relationships with the context and with some of the participants, was contained in the study. The research was also personally interested in the objective of the research. In relation to Domain 2, the methodology used in the design of the study has been widely developed, and the existence of a theoretical framework can be verified; in this case, the framework was chosen in regard to the contrast of “etic” and “emic”, as described by Pike and Harris, and used sampling for convenience, snowball sampling and theoretical sampling to select the participants. The processes of saturating and triangulating the data with primary sources and other researchers are vital; thus, all the transcripts were validated by the informants themselves.

Finally, in relation to Domain 3 [51], the information obtained was coded and categorized following a tree of meanings and topics. The participants, the research team and other researchers validated the information obtained, which gave approval to the consistency of the information obtained. This information was categorized into major and minor topics, which shows that the research was contrasted in a masterly way in the Section 4.

Furthermore, Guba and Lincoln’s alternative criteria are as follows: credibility, dependability, confirmability, and transferability [52].

Credibility: The main research was accompanied by a local team from the Fang ethnic group, in order to counteract the researcher’s perspective or the effects of their presence on the nature of the data. At the end of each day, notes made in the field notebook, participant observations and the interview recordings were reviewed.

Reliability Triangulation was carried out via three different pathways: Data triangulation: the participants were of different ages, from different districts, and had different roles within the Fang community and the therapeutic process. Researcher triangulation was achieved: people who have worked together with the Fang ethnic group in Equatorial Guinea in other contexts, i.e., in the field of education, culture, sports, etc., validated the results. Methodological triangulation was achieved: several data collection techniques were used, including semi-structured interviews, participant observation, the use of a field notebook, etc.

Confirmability. All the interviews were filmed, transcribed verbatim and validated; this was accomplished first by the main researcher, then by the local facilitators, by the informants themselves, who came to modify or complete some expressions, and by an outside member of the Fang community. All of them stated that the gathered information faithfully represents the nature of traditional Fang medicine in Equatorial Guinea.

Transferability. The findings can be transferred to other cultural contexts in Africa in which traditional medicine is more deeply rooted and with which the Fang ethnic group of Equatorial Guinea shares its practices, symbology, and trajectory.

### 2.6. Ethical Considerations

Authorization was issued by the General Directorate of Pharmacy and Traditional Medicine of the Ministry of Health and Social Welfare once the justification report of the project was examined; this included the objectives, methodology, script of the interview, participants, etc., and was a document in which the verbal consent of the participants was stated [53]. In addition, an Ethics Committee called the “Ethic and Regulatory Issues, Clinic Research Projects” of La Paz Medical Center in Bata (a private hospital with international health coverage), comprising the Medical Director, the Head of the Emergency Services, the Head of Internal Medicine Services and a data manager, who are all medical professionals from Israel with proven experience in validating research projects in their country, approved the procedure referred to in the dossier. The delivered documentation consisted of a supporting report, the design, methodology, participants, interview script, and a modified informed consent sheet, which is frequently used in qualitative methodology research projects in which express reference is made to the verbal consent of all the participants. The assent and conformity was recorded [49,54,55].

Finally, the participants themselves listened to the reading of the content of the informed consent sheet, gave their consent verbally, and were offered the possibility of making suggestions once the reading of the text was finished. In total, 40 of the 45 informants could not read or write in Spanish, or had a significant visual deficit, so consent was obtained verbally. They enthusiastically and voluntarily agreed to share the information with third parties and be recorded in their natural habitat. For one or more of the local witnesses, his own family was present when oral consent was granted. They were informed that they could request a copy of the recording at any time.

The research did not entail any risk for the participants, since the proposed methods did not involve a compromise of the physical/psychological/social integrity of the people who participated in it. Common procedures were used for data collection. At all times, the participants were informed that the objective of the research was to find out about the way of life of an ethnic group, particularly in regard to their customs, their representations of disease, and their therapeutic itineraries, etc.; indeed, our interest was far away from making value judgments or exalting one culture over others.

The anonymity of the participants was preserved in the final writing of the report in order to prevent their identification, as well as in the field notes, in case the field notes were required for inspection. Individual entities were described numerically, so that other community members could not identify participants when the findings were shared in order to ensure the accuracy of these findings.

All data collected were also treated and analyzed confidentially. In order to achieve the anonymity of the participants, names and details that could reveal the identity of the participants, any other persons mentioned, and the institutions referred to were removed or adapted from the transcript of the interviews.

The interviews were filmed at home or in the Fang tribal villages; in the case of interviews with elders, some members of the family were present. External facilitators, Fang community leaders, FTHs and healthcare professionals received monetary compensation for the journey to his/her place of residence. The elders received a set of hygiene products and non-perishable food.

All the transcripts were coded. The external facilitators did not participate in the design of the study or in the subsequent processing of the information for analysis [47].

The authors assert that all the procedures that contribute to this work comply with the ethical standards of these committees, and are in accordance with the ethical standards established in the 1964 Declaration of Helsinki and its later amendments.

## 3. Results

### 3.1. Specialties of FTM

FTM can be categorized according to different fields of work. Health professionals and FTHs have differentiated two types of FTM:

The first is FTM that is based on herbs and tree barks that can cure prevalent diseases in the country, such as poisoning, malaria, sexual impotence problems in men, fertility problems in women and bone fractures (the participants referred to the Ndong clan’s entourage), and acute signs or symptoms, such as difficulty breathing, fever, pain, diarrhea or convulsions; the second is FTM that offers protection from misfortunes via therapies for cultural or folk illnesses, such as “eluma” and “witchcraft” (pathological syndromes without a known diagnosis that present with non-specific clinical manifestations in an ethnic group) after contacting with the spirits of dead people using witchcraft and other practices.

### 3.2. FTM: The Learning Process

Several methods for learning FTM were described by the relatives of FTHs and Fang elders as being mainly empirical and without the need for traditional schooling, unlike with Western medical professionals:

FTHs can learn by watching a family member (grandparent, uncle, father, mother-in-law, etc.) who works as an FTH.

“*I learned it from my mother-in-law; she was the healer, and she always sent me to look for medicines to the forest*.”

E51 ESISIS clan, FTH

The spirits or ancestors can reveal healing knowledge to the FTH, such as in the following example:

“*If you have an ancestor, and he maintains a good relationship with you, this person can come in a dream and tell you the remedy*.”

E9 ANVOM clan, healthcare professional

Finally, there are some patients who have spent long periods of time in the hospital or visit a traditional Fang healing post where the FTH has learned how to deal with a certain disease.

### 3.3. FTM: Therapeutic Procedures for Curing Diseases

**Figure 1:**
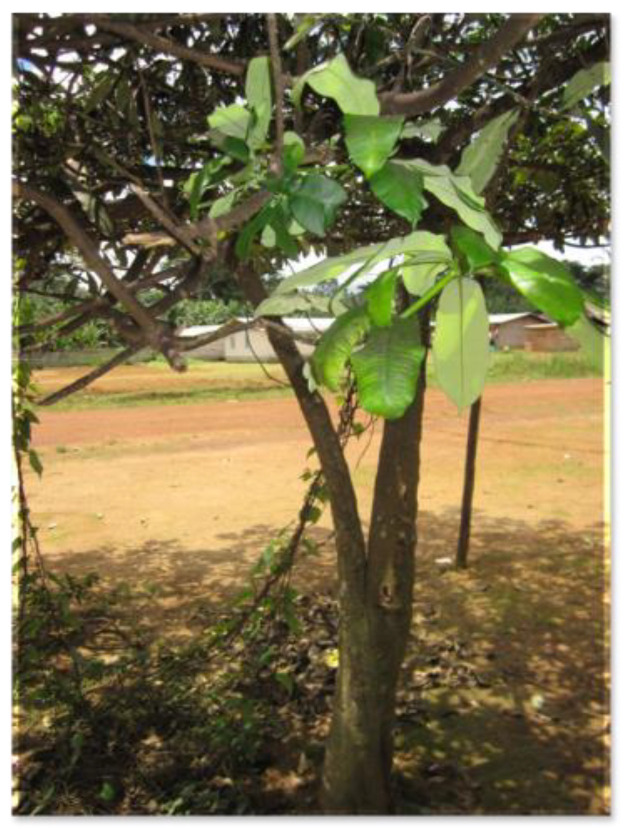
Ekuk tree in Akonibe District.

Materials from forests: tree barks and plants and rituals are used to keep Fang populations healthy (Figure 1). This is shown in the following description:

“*When a child is circumcised, we apply ‘ndong;’ we chew the seeds, and with saliva, we spit on the wound, and it heals*.”

E53 OBUK clan, FTH

“*[We use] ‘ekuk’ roots with palm oil, so that the placenta comes out.” “The ‘nfoo’ tree has an effect against a type of malaria*…”

E51 ESISIS clan, FTH

“*The delivery will go faster, if we give them hot water with the roots of ‘okum’*…”

E35 ESAVENG clan, FTH

“*‘Abehe’ is the fruit of a tree that serves to cure impotence in men; also ‘akak’, taken by men, gives them strength*.”

E34 ANVOM clan, FTH

“*If you eat the fruit of a tree called ‘enie’, it is very poisonous; past people wiped it on their arrowheads to hunt. Also, the bark of the ‘esia’ has some poison; women avail it themselves to abort*…”

E29 ESENG clan, FTH

Another practice conducted by the FTHs is animal sacrifice using domestic animals, such as goats and chickens, to obtain their blood and add it to their preparations. The sacrifices are used as payment to the spirit that connects the FTH with the world of the ancestors. ‘Etoak’ is well known to FTHs as a container in which herbs, tree bark and, sometimes, animal blood are mixed. The mixtures are placed as a topical ointment on the part of the body that patients indicate discomfort. Predictive information about the origin of various diseases is taught to the FTHs.

Finally, some of the interviewed elders highlighted a ritual known as ‘osuiñ’, in which the patient confesses his or her sins in the river in the presence of the FTH. If the patient does not admit his or her guilt, the FTH can force a confession using herbs or hallucinogenic substances, such as “iboga”, to elicit the confession.

Other therapeutic procedures that were said to occur in traditional medicine posts during the interviews and that were verified by the healthcare professionals and elders, but that were not mentioned by the FTHs, included scarification (when superficial skin wounds made with razor blades and then traditional treatments are applied so that the healed skin will simulate a tattoo), the immersion of patients in boiling water to scare away the “evú”, and the blowing of fire towards a patient wrapped in large banana leaves.

### 3.4. Social Image of FTHs

The social image of FTM described by the participants ‘*is not positive’* E38 YEMBAN clan, Elder. FTM is viewed as a business outside the law, and there is little confidence in its effectiveness in managing the course of a disease. The participants insisted that there had been a substantial change in mentality with the passage of time: whereas before, FTHs worked more with nature, ‘*…nowadays, things have changed; [FTM is] a means of “illicit enrichment” …*’ E43 OBUK clan, healthcare professional.

Furthermore, ‘*the population do not trust the traditional doctors of today, within the family; the Fang healers originally worked with natural products, and today’s healers include some evil practices…*’

E48 OYAK clan, Fang community leader.

Some of the healthcare professionals surveyed noted that patients may spend long periods of time at traditional Fang medicine posts, but sometimes to no avail. In addition, the exact doses or duration of treatments, their methods of evaluation, and the certification of their procedures are not known. Healthcare professionals confirm a lack of scientific-technical competence and basic knowledge about physiology, anatomy, and physiopathology among FTHs, which translates, in some cases, into iatrogenesis and even death. At the hospital, nevertheless, they work with FTHs to formulate criteria for referring patients to Western medicine systems and evaluating the efficacy of the healing products collected from nature.

The interviewed elders reported that the Fang population in EQ exhibits a great deal of respect for FTHs who continue curing diseases with ancestral knowledge, but FTM has been distorted. They disagree with new healers who handle hallucinogenic substances and attribute the possible origin of diseases to witchcraft that is based on moral, familiar, social and spiritual perspectives, rather than based on the etiology of the disease. To the elders, FTM looks like a cult, but the interviewed participants believe that it is not proliferating among youth.

### 3.5. Traditional Medicine User Profiles

FTHs believe that their primary customers are women and those without financial means who can pay in kind with animals, fruits, and vegetables The first step is advertising signs of one’s illness to one’s family. The elders told us that there is one traditional Fang medicine post for each village, and villagers are also more prone to visit the FTM if they closely follow traditional rules. Finally, those who are short on money and want to protect their status visit FTMs.

## 4. Discussion

Through diverse perspectives, this study explored the informal health-seeking behavior of the Fang population in mainland EQ for medical or cultural afflictions. No significant differences were found among the discourse of the participants, traditional leaders, elders, and FTHs of different clans and districts with respect to the studied categories. They all share similar accounts in this study’s analysis, and even the healthcare professionals were familiar with the practices and procedures of FTM in EQ.

Our study shows that FTM is used to handle two types of prevalent diseases: those whose origin is psychological, such as mental illnesses [14,15], and those involving physical health, such as malaria [16,17], difficulty breathing, sexual reproductive health [18], bone fractures, and poisoning, as well as cultural afflictions. The specialization of tasks in FTM has been found in the literature, as well as among other African and Latin American ethnic groups. For example, in South Africa, in the KwaZulu-Natal region, Zuma et al. [56] identified a similar classification between the “isangoma”, fortune-tellers and the “inyanga”, those who work with traditional remedies involving herbs, trees and animals.

In Venezuela, Wayuu healers have described the existence of different types of healers: “outshi”, who attend to common diseases; “emeijut”, who are specialists in the delivery of babies; the “oulakut”, who work in the field of divination; and the “anaajüt jipu”, who are specialists of the skeletal system [57].

The data from the study suggest that the most common method of training in FTM is via the transmission of knowledge about the management of diseases and therapeutic products from generation to generation within the family. For the Fang community, the clan and the tribal village are the engines of individual learning [15,58].

Most of the time, their relatives work as assistants during the learning process, preparing ointments or looking for ingredients in the forest [59].

In one famous Fang family, the Ndong clan, the adult men used to learn the practice of bone setting. In Ogwa (Nigeria), there is a family, the “Idunmunkpaghan,” whose adult men are all engaged in the traditional healing of bone fractures [27,60].

One previous study also indicated that in Nigeria, both in the Igbo ethnic group [55,61] and the Yoruba ethnic group [62], the grandchildren accompany their grandparents during the collection of medicinal plants and during the treatment process.

Another path to becoming a true FTH is by receiving guidance from the spirits, who transmit the knowledge of how to treat diseases. FTHs on this path derive their skills from their ancestors [23,63]. Similarly, Ojibwe traditional healers explain that they are inspired through dreams and that their powers come from the Great Spirit. The spirits guide them on the right path when a patient presents with a disease [64].

The third method of becoming a healer, described by the elders and FTHs alike, involves healers spending long periods of time in traditional healing posts as patients, observing the procedures and learning how to treat certain diseases. Traditional healers in Mexico City, especially those who treat spiritual problems, also learn their practice this way [65].

Finally, FTM based on herbs and tree bark requires more learning time than the magic = religious medicine used by spiritualist healers. Zuma T. et al. [56] support this finding, as they indicate that there are different learning paths taken depending on the healer’s intended specialty. In addition, the author’s discuss that it is a matter of gender, as women learn about issues related to women’s health from their mothers and grandmothers, while uncles and fathers train male children in their own traditions [19,28,58].

Our findings show that FTHs use two main healing techniques based on their own cultural beliefs about the particular illness [14]: Biological causes require medicinal techniques involving herbs and trees, while cultural or supernatural causes [59] call for techniques that involve witchcraft or similar sources of healing that integrate spiritual powers, magic or esoteric rites. Among the medicinal plants mentioned by the participants, both by FTHs and others, we found that tree bark is known for its therapeutic effects and is used in FTM posts: “*ndong*” is known as *Aframomum melegueta* L. *Schum*, has healing and anti-inflammatory properties [20,66] and is used by the Obuk clan. The “*esiá*” tree is used by the Eseng clan to induce abortion and is known in the pharmacopoeia as *Tephrosia vogelii Hook* [66].

“*Ekuk*” is widely known among Fang communities, and palm oil is useful for expelling the placenta during childbirth. The plant is described as *Alstonia boonei de Wild*, and it also helps with the antipyretic effects of malaria [21,24,25,67]. The Botanical Garden of Madrid, after having sent a research group to EQ in 1989, found great similarities among the narratives of the participants [4,24,25,66,67,68].

In addition to the use of plants and tree barks, the FTHs reported that they add the blood of sacrificed domestic animals to their preparations, which is insufficiently described in the literature. Our study confirmed that the sacrifices represent a tribute that must be paid to the spirits, either to the spirit of the healer for having contacted the ancestors, or as food for the spirit of the wounded or sick. For the Fang community, the spirits of animals and the souls (“*evú*”) of humans are connected [69].

Traditional healers of the Awori ethnic group in Nigeria have also used animals for religious or cultural purposes to invoke spirits and gods [70]. Pangolin scales are highly prized in other countries in Africa [3]. In the case of Wayuu healers in Venezuela, a tribute is negotiated with the spirits to rescue the soul of the sick, with the spirits asking for material goods or animal sacrifices in return [57].

To conclude, the participants described the importance of the patient’s confession of faults and mistakes made in the river, the “*osuiñ*”, alongside the FTHs. The Fang population considers illness to be a punishment for sin or breaking traditional rules, such as eating forbidden animals or becoming engaged to a woman of the same clan origin [10]. For the Fang, one’s good health is the outcome of a perfect harmony between humans and their context [8].

In Nigeria, healers also conduct rituals in designated places in the forest, such as the dwelling places of their ancestral spirits [8]; the technique of confession was also used by the Mayans when diagnosing a disease whose origin was the breaking of a rule [71].

FTHs sometimes combine this procedure with an “*etoak*”, a container that is placed on the area in which the patient is experiencing discomfort. Often, it provides diagnostic information on the health problem affecting the patient. In Nigeria, traditional healers employ a similar technique that involves looking through a mirror or a pot full of water, and observing the events that led to the patient’s current state of illness [8].

The repertoire of traditional healers in Tanzania also includes massages that inflict burns with water, hot stones or steam baths [72]. For curing tuberculosis, traditional healers in Mexico use “*sopladas*”, where the healer takes brandy, warms it in his mouth, and then blows it onto the body of the patient [73]. Traditional healers in Zambezia (Mozambique) used to inoculate patients using subcutaneous preparations in superficial wounds made with razors on the back and shoulders [24].

Our findings suggest that the Fang population respects FTM; FTHs speak the same language or use the same explanatory model of illness as the patients [74], which gives the latter a sense of comfort and well-being. Patients seek relief from stress attacks that are generated by the disease, and act as intermediaries between life and the ancestors in death [75]. The high demand for FTM has led some healers to see such practices as a very lucrative business [72]. Today, FTM knowledge has been accused of being a false ‘tradition’ [26].

Furthermore, there has been a return to ancestral medical knowledge even though the knowledge has deteriorated, partly due to there being limitations on information access and partly due to the scarcity of true healers. However, at the same time, this practice has grown, as evidenced by the influence of religious medicine. In a study published in Tanzania, Kayombo et al. [72] concluded that health workers consider methods used by traditional healers to be far from scientific; situations such as childhood fever or malaria are confused with traditional beliefs and superstitions associated with witchcraft, resulting in improper treatment. In line with other studies in Africa, some biomedical perceptions of TM suggest that healers’ practices are underdeveloped or “backward” [30].

In our study, a lack of control or certification of the procedures used by FTHs was documented. In Cameroon, one of the problems associated with the use of herbs for treatments is that some of them have never been evaluated or standardized [76].

In addition, there is a lack of basic knowledge among FTHs regarding anatomy, clinical pathology, etc., which can result in iatrogenic events and even death [77]; the referral of patients between therapeutic resources is very scarce and often too late [78,79]. The work of bone setters is widely described in the scientific literature. In Nigeria, the development of gangrene is a common adverse result of the techniques used by traditional healers to heal fractures [29], with 31.8% of amputations leading to gangrene as a result of treatment by FTHs [77].

Finally, the profiles of traditional medicine users are influenced by socio-economic factors [59]: difficulty obtaining cash; the implementation of a barter system; a lack of healthy fruits, vegetables or animal meat; and the lack of other local therapeutic resources, especially in the case of undertreated mental illness in EQ. These results are in agreement with those of other studies conducted in African countries. Furthermore, as found in other studies in Africa, the majority of those who seek health resources similar to FTM are female, who already carry the family burden [17,58,80], or elders, who are devoted to their cultural beliefs [81].

## 5. Limitations

Current research conducted with methodological rigor on the traditional medicine of the Fang ethnic group has not been found in the literature. It is a topic scarcely explored in the literature, probably due to the difficulties in accessing information, obscurantism, the fear of intrusion on or plagiarism by healers, and the supposed supremacy of biomedicine, despite FTM being a resource of choice in the population of Sub-Saharan Africa. In Equatorial Guinea also, knowledge transfer about traditional medicine does not take place appropriately due to the lack of interest among the younger generation. It is likely that when the participants of this research die, knowledge will be lost.

This study focuses on four perspectives: elders, traditional healers, healthcare professionals and community leaders; we did not gather from other different Fang tribe providers. The analysis validation and publication of this study were delayed due to the arrival of COVID-19 and the restrictions imposed on travel, local, national and international (2020–2021).

Finally, when the complex procedures of transcribing, translating, and conceptualizing metaphors, concepts, and Fang symbology, etc., were completed, validating and analyzing the data demanded extra effort, so as to preserve the literal meaning of the message.

## 6. Conclusions

FTM continues to play a relevant role in the process of curing diseases among the Fang community in EQ. Elders who are close to traditional rules and women who can pay in kind are the primary users of FTM; young people are less likely to use FTM, which should be further studied in the future. FTM practitioners work empirically, and they learn from their relatives or from the spirits of their ancestors.

Malaria, poisoning, reproductive health, and folk illnesses can be treated by FTM, and the findings suggest that the informal health-seeking behavior among the Fang community is conditioned by the explained model of illness.

Fang populations have a negative social image of FTHs, and sometimes perceive them to be driven by economic business rather than a desire to heal. There has been a boom of so-called spiritual healers who have distorted the true essence of traditional medicine with the introduction of new therapeutic resources, such as scarification, immersion in boiling water and blowing fire on patients.

In addition, it is recommended that the range of quality choices for other therapeutic procedures and state healthcare services be provided free of charge.

The greatest challenge of the WHO agenda 2014–2023 in EQ will be enhancing institutional capacity, with a particular focus on monitoring and assessing the uses and properties of medicinal plants, training stakeholders, and enacting legal frameworks for traditional healthcare practice. The agenda should help create possibilities for collaboration among the practitioners of FTM and other curative options.

## Figures and Tables

**Table 1:** Categories of participants (n = 45).

Category	n	%
Tribal Elders	19	42.2
Community leaders	6	13.3
Healthcare professionals	7	15.5
FTH Fang Traditional healer	11	24.6
Traditional healers’ relatives	2	4.4
Total	45	100

**Table 2:** Fang tribes and category of participants (n = 45). Esawong Tribe.

Fang Tribe	Code	Category
Esawong Tribe	E2	Fang Traditional Healer
	E13	Healthcare Professional
	E14	Community Leader
Esanwang Tribe	E1	Traditional Healer’s Relatives
Oserengon Tribe	E7	Tribal Elder
	E19	Tribal Elder
	E24	Healthcare Professional
	E46	Tribal Elder
Esanvus Tribe	E18	Tribal Elder
Onvang Tribe	E8	Fang Traditional Healer
	E40	Community Leader
	E45	Healthcare Professional
Anvom Tribe	E28	Tribal Elder
	E10	Community Leader
	E9	Healthcare Professional
	E26	Community Leader
	E58	Tribal Elder
	E34	Fang Traditional Healer
Yesuk Tribe	E17	Tribal Elder
	E27	Tribal Elder
Efak Tribe	E22	Tribal Elder
Yemban Tribe	E38	Tribal Elder
Yemandjim Tribe	E39	Tribal Elder
Yenken Tribe	E56	Tribal Elder
Ngama Tribe	E57	Community Leader
Esumu Tribe	E32	Tribal Elder
Mbon Tribe	E23	Tribal Elder
Esisis Tribe	E51	Fang Traditional Healer
Eseng Tribe	E29	Fang Traditional Healer
Esakora Tribe	E55	Tribal Elder
Nsomo Tribe	E25	Tribal Elder
	E44	Healthcare Professional
Angok Tribe	E33	Tribal Elder
Ncodjen Tribe	E16	Traditional healer’s Relatives
	E20	Tribal Elder
Ndong Tribe	E41	Fang Traditional Healer
	E42	Fang Traditional Healer
Oyak Tribe	E48	Community Leader
	E49	Tribal Elder
Esaveng Tribe	E35	Fang Traditional Healer
Obuk Tribe	E43	Healthcare Professional
	E47	Healthcare Professional
	E50	Fang Traditional Healer
	E52	Fang Traditional Healer
	E53	Fang Traditional Healer

**Table 3:** Data collection: Fang Tribal villages.

District	Fangtribalvillage
Mongomo	Noankien, Onvang-Etom, EnukEsawong, Mbon-Ngolom
Akonibe	Bisobinam, Engong, Ebom, Afanam
Ebebiyin	Ntu-Angok, Alen-Angok
Niefang	Dum, AlenCdo, Ayene, Dumasi
Kogo	Nkoho, Nkoamben, Río Muni, Basile, Babe, Asuiabee, Ondeng

**Table 4:** Socio-demographic profile of participants (n = 45).

1. Socio-demographic profile of participants (n = 45)			
**Concept**		**n**	**%**
**Gender**			
Female		8	17.8
Male		37	82.2
Total		45	100
**Age**			
21–30		1	2.2
31–40		0	0
41–50		4	8.9
51–60		6	13.3
61–70		16	35.5
71–80		11	24.6
81–90		5	11.1
91–100		2	4.4
Total		45	100
**1.1. Socio-demographic profile of FTH (n = 11)**			
**Concept**		**n**	**%**
**Gender**			
Female		2	18.1
Male		9	81.9
Total		11	100
**Age**			
41–50		1	9.1
51–60		4	36.3
61–70		2	18.2
71–80		2	18.2
81–90		2	18.2
Total		11	100
**2. Socio-demographic profile of participants (n = 45)**			
**Concept**		**n**	**%**
**Marital Status**			
Married		12	26.7
Married polygamous		28	62.2
Widows		4	8.9
Singles		1	2.2
Total		45	100
**3. Socio-demographic profile. Household data of participants (n = 45)**			
**Concept**		**n**	**%**
**Residence**			
Capital Region		5	11.1
Capital Province		6	13.3
Capital District		6	13.3
Fang tribal village		28	62.3
Total		45	100
**District**			
Mongomo		10	22.2
Kogo		11	24.4
Ebebiyin		5	11.1
Akonibe		7	15.6
Niefang		8	17.8
Bata		3	6.7
Mbini		1	2.2
Total		45	100
**Main Material of the Exterior Wall**			
Wood planks		21	46.7
Cement blocks		24	53.3
Total		45	100
**Household Facility**			
Electricity	Yes	33	73.3
	No	12	26.7
Total		45	100
TV	Yes	33	73.3
	No	12	26.7
Total		45	100
Refrigerator	Yes	34	75.6
	No	11	24.4
Total		45	100
Radio	Yes	44	97.8
	No	1	2.2
Total		45	100
Vehicle	Yes	13	28.9
	No	32	71.1
Total		45	100
**3.1. Household data of traditional healers (n = 11)**			
**Concept**		**n**	**%**
**Residence**			
Capital Region		1	9.1
Capital Province		2	18.2
Capital District		1	9.1
Fang tribal village		7	63.6
Total		11	100
**District**			
Mongomo		3	27.3
Ebebiyin		2	18.2
Kogo		1	9.1
Akonibe		4	36.3
Bata		1	9.1
Total		11	100
**Main Material of the Exterior Wall**			
Wood planks		7	63.7
Cement blocks		4	36.3
Total		11	100
**Household Facility**			
Electricity	Yes	8	72.7
	No	3	27.3
TV	Yes	8	72.7
	No	3	27.3
Refrigerator	Yes	9	81.8
	No	2	18.2
Radio	Yes	11	100
	No	0	0
Vehicle	Yes	4	36.3
	No	7	63.7
	No	6	54.5

**Table 5:** Categories (cat.) and Subcategories (sub). Analysis.

**Cat. 1. Specialties of Fang Traditional Medicine (FTM)**	Subcat.1. Curing prevalent diseases, based on herbs and tree bark
	Subcat.2. Therapies for cultural folk illnesses
**Cat. 2. FTM: The Learning Process**	Subcat.3. Watching a family member
	Subcat.4. Spirits and ancestors reveal healing knowledge
	Subcat.5. Now patient in the healing post, then traditional healers
**Cat. 3. FTM: Therapeutic Procedures for Curing Diseases**	Subcat.6. Materials from forests: tree barks and plants
	Subcat.7. Etoak: Animal sacrifice
	Subcat.8. Osuiñ: confessing his/her sins in the river
	Subcat.9. Other therapeutic procedures: scarification, immersion into bowling water.
**Cat. 4. Social Image of FTHs**	Subcat.9. It is not positive
	Subcat.10. Lack of scientific–technical competence
	Subcat.11. Great deal of respect for FTHs
**Cat.5. Tradtional Medicine User Profiles**	Subcat.12. Women
	Subcat.13. People without financial means
	Subcat.14. Villagers closely to the traditional rules

## Data Availability

The datasets used and/or analyzed during the current study are available from the corresponding author on reasonable request.

## Abbreviations

FTM	Fang Traditional Medicine
FTH	Fang traditional healer
TM	Traditional medicine
EQ	Equatorial Guinea
WHO	World Health Organization
ASOMETRAGE	Traditional Medicine Association in Equatorial Guinea
UNDP	United Nations Development Program
HDI	Human Development Index
INB	Gross national income per capita
GDP	Gross domestic product
INSESO	National Institute of Social Security
FERS	Spanish Federation of Sanitary Religious
DHS	Demographic and health surveys
COREQ	Consolidated criteria for reporting qualitative research
